# A New Labeling Approach for Proportional Electromyographic Control

**DOI:** 10.3390/s22041368

**Published:** 2022-02-10

**Authors:** Annette Hagengruber, Ulrike Leipscher, Bjoern M. Eskofier, Jörn Vogel

**Affiliations:** 1 German Aerospace Center (DLR), Institute of Robotics and Mechatronics, 82234 Weßling, Germany; ulrike.Leipscher@dlr.de (U.L.); joern.vogel@dlr.de (J.V.); 2 Machine Learning and Data Analytics Lab, Department Artificial Intelligence in Biomedical Engineering, Friedrich-Alexander-University Erlangen-Nürnberg (FAU), 91052 Erlangen, Germany; bjoern.eskofier@fau.de

**Keywords:** electromyography, human machine interface, robotcontrol, EMG-control schemes

## Abstract

Different control strategies are available for human machine interfaces based on electromyography (EMG) to map voluntary muscle signals to control signals of a remote controlled device. Complex systems such as robots or multi-fingered hands require a natural commanding, which can be realized with proportional and simultaneous control schemes. Machine learning approaches and methods based on regression are often used to realize the desired functionality. Training procedures often include the tracking of visual stimuli on a screen or additional sensors, such as cameras or force sensors, to create labels for decoder calibration. In certain scenarios, where ground truth, such as additional sensor data, can not be measured, e.g., with people suffering from physical disabilities, these methods come with the challenge of generating appropriate labels. We introduce a new approach that uses the EMG-feature stream recorded during a simple training procedure to generate continuous labels. The method avoids synchronization mismatches in the labels and has no need for additional sensor data. Furthermore, we investigated the influence of the transient phase of the muscle contraction when using the new labeling approach. For this purpose, we performed a user study involving 10 subjects performing online 2D goal-reaching and tracking tasks on a screen. In total, five different labeling methods were tested, including three variations of the new approach as well as methods based on binary labels, which served as a baseline. Results of the evaluation showed that the introduced labeling approach in combination with the transient phase leads to a proportional command that is more accurate than using only binary labels. In summary, this work presents a new labeling approach for proportional EMG control without the need of a complex training procedure or additional sensors.

## 1. Introduction

Human machine interfaces based on electromyography (EMG) are a technology used in many different applications. Besides the control of prosthesis, which is the most common application of EMG control, it is nowadays also used in rehabilitation [1], robot control [2], in computer gaming interaction [3], or for teleoperation in space applications [4].

Depending on the application, different control strategies are realized to use the voluntary muscle activity as an input signal for an external device. In commercially available prosthesis conventional control techniques, such as threshold-based methods [5,6], or linear classification [7] are still widely used. However, usually just a few degrees of freedom (DoFs) are sequentially controlled by these techniques. More complex devices, such as a robot or multi-fingered prosthesis, require a more natural and versatile control scheme. Therefore, proportional and simultaneous control strategies are becoming more widely adapted.

While proportional control allows the user to continuously change the control output by varying the control input, i.e., the EMG signal, simultaneous control further enables the user to command multiple available motor functions or DoFs of the system at the same time [8]. One way to realize proportional control with classification is given by Simon et al. [9], where the authors present a two-step method. First, a classifier is trained to differentiate between the classes. Second, the mean absolute value (MAV) of all EMG channels per class is applied to calculate a continuous control output. However, this method only allows to control one motor function or class at a time. To overcome the problem of non-simultaneous control, regression can be used instead of classification. Regression allows for a continuous output for multiple DoF and thereby enables simultaneous control. Hahne et al. [10] could further show that regression leads to an improvement in performance and also allows for a better user correction of control commands, when comparing regression to classification in an online test. Various machine learning methods have been applied to realize regression methods with EMG. For example, artificial neural networks (ANN) are commonly used [11,12,13], as well as support vector regression [14], or more recently convolutional neural networks [15]. To realize proportional control, the machine learning methods are also often combined with continuous data gathered by additional sensors. One example for this is given by Castellini et al. [16], where EMG signals have been combined with force measurements to control a dexterous multi-fingered hand.

Independent of the mapping method, proportional control methods come with typical problems of myocontrol, such as sensor shift, signal drift, or muscle fatigue [17,18]. On top of that, these methods exhibit the additional challenge of acquiring a suitable training data set with correct labels for model building. Within the last few years, different research groups investigated a variety of methods to provide suitable training data sets for proportional control. A quite common way to generate the labels is to ask the user to track a visual stimulus on a screen. The stimulus can be illustrated, for example, by a moving cursor or the motion of an animated or video-recorded hand shown on a computer screen such as in [19,20,21]. EMG data are recorded while the user is following the stimulus on the screen and the label is calculated based on the state of the visual stimulus. An alternative way to generate the labels is to use additional tracking devices such as a camera [13,22] or data glove [23]. Here, the EMG signal is fitted to the motion gathered by the additional sensors during the training procedure. In terms of amputees, the contra-lateral hand/arm can be tracked with the sensors while EMG signals are recorded from the arm used with the prosthesis. This method is called mirroring. EMG signals are also often interpreted as force. Therefore, additional force sensors can be used to generate the training data set. Users exert, for instance, finger force to a force sensor during the training procedure [24].

Additional sensors, such as force sensors, measure the motor output directly, which can be considered as the ground truth of the EMG decoding, which makes it the optimal signal to generate labels for the mapping. However, in terms of people with disabilities, additional sensors are usually not an option. People with motor impairments often have a limited proprioception of muscular activity and in case of amputees, finger force can not even be measured [24]. In addition, mirroring can cause wrong data, as it is hard for the subjects to provide exact mirror movements. In Hahne et al. [25], the performance of hand movements dropped when using labels generated with the co-lateral hand in comparison to that of the ipsy-lateral hand.

Generating labels without additional sensors, e.g., using a visual stimulus, comes with the challenge to synchronize the EMG signals to the data used for the labels. When generating EMG activation during the training procedure, the user may lag behind the visualization on the screen. Poor synchronization of the data can lead to mismatches between EMG data and labels, which may result in unreliable or simply wrong control commands during usage. This can lead to unintended movements of the remote device and therefore to frustrated users [26].

An additional challenge for the training data set used in proportional EMG control is to gather the required variations in EMG signals in correlation with the desired continuous control output. A relevant influencing factor for a robust EMG-based control lies in the in- or exclusion of the transient phase. Fougner et al. [8] state that the training data needs to be as realistic as possible, including continuous movements to achieve good proportional control. The transient phase of an EMG signal is defined as part of muscular activity in which the signal rises from rest to contraction level. It includes the burst of the sudden muscular activity in which not all motor-units (MUs) are activated yet. Compared to this, the steady-state phase is defined as the phase of a constantly maintained muscle contraction [27]. Although Englehart et al. [28] showed in an online study that the usage of steady-state data leads to more accurate performance than using transient data, the transient phase can give information for continuous labeling. Kanitz et al. [21], for example state that the onset of a muscle contraction gives predictive information about the upcoming class. According to Raghu et al. [29], the inclusion of transient data is not trivial, as the segmentation, synchronization of additional sensors, as well as labeling is challenging with this dynamic data. As a result to all these challenges, the transient phases of the signal are often omitted, while the steady-state phase is regularly used for training, as, for example, shown in [30].

Nevertheless, literature presents various possibilities to generate continuous labels with varying EMG data. One option is to record and label EMG signals in a graded representation. Therefore, the user provides muscular activity with different levels of activation, e.g., at a low, medium, and high level, depending on the maximum voluntary contraction (MVC) during the training procedure [31]. Another option is to continuously ramp the motion during training from no contraction to a defined contraction level [32,33]. In Hahne et al. [10], the subject had to increase intensity up to 80% of MVC while a cursor on the screen moved along the axis in order represent the required intensity. Zia et al. [34] asked the subjects to provide muscular activity with contraction and relaxing periods of 4 s. In Jiang et al. [35], the forces, which had to be produced during the training procedure, were visualized on a screen. Subjects were asked to ramp muscle contractions to a medium force level which was recorded with a force-torque sensor. The force levels were then used as labels. Gailey at al. [36] also used force sensors to measure finger force during the training procedure. Phases of in- and decreasing forces allow one to measure different force levels corresponding to the EMG data stream.

In this work, we focus on an EMG-based interface designed for robot control. The main application is for people suffering from muscular atrophy. The interface maps available residual muscular activity to a velocity-based output by using Gaussian process regression. As presented above, generating continuous labels for proportional control is often associated with the effort to fuse data from multiple sources. In terms of people with disabilities, this is often not an option. Furthermore, complex training procedures are often used to generate the labels, which can cause unreliable control commands.

The interface we are using provides a proportional output and thus comes with the given challenges. In this work, we address these challenges and introduce a new labeling approach to generate continuous labels for proportional control in an easy and direct way. The introduced method directly uses the EMG feature stream of the training data and creates continuous labels without the use of any additional sensors. As the label is calculated directly based on the feature stream of the EMG signal, delays are reduced to a minimum. Furthermore, a simple training procedure leads to easy application for users. We compare the method to a standard binary labeling method and investigate the effect of the different labeling strategies on task performance when operating a continuous and simultaneous EMG-based interface. Additionally, the influence of in- and excluding the transient phase of the EMG signal is analyzed.

To validate this approach experimentally, a user study with 10 subjects was conducted using the velocity-based EMG interface. The task performance was analyzed with the help of a 2D aiming and tracking task on a screen.

To summarize, the contribution of this work is to introduce a new labeling approach, which generates continuous labels for proportional control in an easy and direct way. Furthermore, the work includes a validation of this new labeling approach during task performance and the effect compared to a binary labeling approach.

## 2. Materials and Methods

### 2.1. The EMG-Based Interface

**Figure 1:**
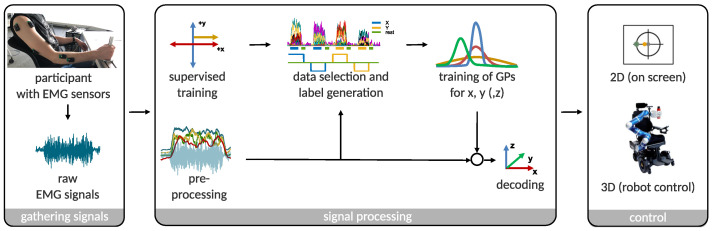
A schematic overview of the EMG-based interface used in this study. **Gathering signals:** A participant is shown with the EMG sensors, holding a handle to generate isometric muscle contractions. Raw EMG signals are wirelessly transferred for further processing. **Signal processing:** Four time-domain features are calculated from the raw EMG signals. A supervised training procedure allows to generate the labels for the prediction. The labeled data is mapped to a velocity-based control command by the use of Gaussian process regression. **Control:** The interface realizes a proportional and simultaneous control output. 2D as well as 3D applications are feasible, e.g., for a commanding an assistive robot. In this study, only 2D control was realized to perform tasks on a screen.

The used interface is interpreting the muscular activity of an operator to control a remote device, e.g., a robot or cursor on a screen. Muscular activity is measured using surface EMG sensors at different locations on the operator’s upper and lower arm. Based on these EMG signals, the interface generates a continuous and velocity-based control signal in 2D or 3D. The main use case of this interface is for people with severe muscular atrophy. It provides people with the possibility to control a robot in 3D, when the usage of a joystick is not an option anymore [37]. An assistive device like the robotic wheelchair EDAN can present such a system, which is commanded via EMG signals by people suffering from severe muscular atrophy (c.f. Figure 1 on the right) [38]. Depending on the user, the sensors are either placed on the prominent muscle bellies, or in case of users with muscular atrophy on spots along the arm where muscles can be still voluntarily activated. In this study, a 2D control input was generated to perform tasks on a screen. The schematic overview of the interface is given in Figure 1.

### 2.2. Experimental Setup

In this work, eight wireless electromyography Trigno*®* sensors from the company Delsys were used to record muscular activity. A medical grade double-sided tape allows for an easy attachment of the sensors to the surface of the skin. To record hand and wrist activity, electrodes were placed close to the muscles M. flexor digitorum superficialis, M. flexor carpi radialis, M. extensor carpi radialis, and M. extensor digitorum, respectively. Two sensors were attached to the upper arm, i.e., the M. biceps brachii and to the M. triceps brachii, and two to the M. deltoid (anterior and posterior). For each participant, the sEMG electrodes were placed on the same physiological spots along the dominant arm. Figure 1 on the left shows a subject wearing the eight EMG sensors. The raw biosignals were amplified and wirelessly transferred to the Delsys Trigno*®* base station. An analog-to-digital converter of the company Beckhoff digitized the ±5 V analog signal from the base station into a 12-bit signal at a rate of 1 kHz. A linux real-time computer received the data via EtherCAT, where the signal was further processed with 1 kHz. The time domain (TD) feature set was used for preprocessing, which was originally proposed by Hudgins et al. [39] to classify myoelectric patterns for the control of a multifunction prosthesis. This feature set includes: waveform-length, slope sign-change, zero-crossing-rate, and sEMG-amplitude. All features are calculated on each EMG channel with a sliding window of 150 samples.

Gaussian process (GP) regression was used to map EMG data to the directional control command (±*x*, ±*y*). Here, the *pyGP* library [40] was utilized, which is based on the implementation of [41]. Participants were asked to exert forces and torques against a rigid handle, which was placed in front of them. Thus, they were able to generate reproducible muscle contraction in an isometric fashion.

### 2.3. The Training Procedure

A training procedure was conducted with each participant at the beginning of an experimental session, in order to acquire data to be used for calibration of the GP. Therefore, participants hold on to a handle with their dominant hand (the same side on which the electrodes are placed) in front of them with an angled elbow. First, the rest signal of the arm was recorded while grasping the handle in a comfortable position without any specific muscle contraction. EMG signals were recorded during this rest state to determine and remove the signal’s DC offset. Furthermore, this rest data allowed to define an individual activity threshold to distinguish between rest and voluntarily activated muscles. Based on this activity threshold, a supervised training procedure was performed in order to generate a training data set for the GP.

In total, four different directions are decoded: left and right (±*x*), up and down (±*y*). To do so, visual cues on a screen guided the participants through the training procedure. The screen displayed a coordinate system illustrating these four directions. One direction at a time was highlighted and participants were asked to exert forces and torques against the handle to be associated with motion along this highlighted direction. A marker moving along the axis and into the direction of interest served as a progress bar, to visualize the amount of collected active samples. Samples were counted as active as soon as the activity threshold was exceeded. Only these active samples were considered as potential training data for the direction. Thus, the activity signal allows to track the users state of participation during the training and the effect of the reaction time is eliminated.

Subjects were asked to provide muscular activity at a level they are comfortable with and stay within the steady-state phase for at least 3 s. They were not asked to ramp the EMG data or reach a special activation level. During data acquisition, raw EMG signals and features were recorded in combination with the indicated direction of motion and the information of an exceeded activity threshold. For each direction, the visual cue was displayed until 3000 active samples have been collected. However, participants had no feedback about their activation level or which muscles were activated during muscle contraction. Once 3000 active samples had been recorded for the requested direction, subjects had to return to a resting state (i.e., stay below the activity threshold). Once remaining in rest for 1000 consecutive data samples, the next direction of motion would be indicated to the subject, until data had been collected for all four directions.

This data acquisition procedure was repeated four times, while only the last three repetitions were used to build the training data sets used for mapping. Gaussian process regression was used to decode continuous velocity commands for each DoF, i.e., one GP for ±*x*, and one for ±*y*, respectively. Further details about the decoder pipeline are given in Vogel et al. [42].

### 2.4. Labeling Approach

The goal is to create continuous labels based on the simple data acquisition procedure described above and without additional sensors. The preprocessed EMG signals, gathered during the training procedure, serve as the basis for the continuous labels. The onset of activation was determined by the activity threshold measured during the rest state and can be used as the onset of the labels. Therefore, unwanted delays due to the participants reaction time can be avoided. However, an intrinsic delay of 47 ms is introduced by the data acquisition system. Furthermore, the feature extraction, which uses a 150 ms sliding window results in additional delay. However, these delays are present not exclusively during training but they also occur during usage of the interface.

In previous work [42], an offline analysis was conducted to reveal the features with the most information content used for the decoding. The results indicated that all four TD features (waveform-length, slope sign-change, zero-crossing-rate, and amplitude) are involved in the prediction. Hence, all four features were used to maintain the influence of the individual features. Furthermore, the data of all eight sensors were considered for the labels, since each directional command is a composition of different sensors. In total, a data stream of 32 features (4 features and 8 electrodes) was processed per time step. All of the following calculation steps have been done separately for each direction.

In a first step, all eight signal values *x* of the eight electrodes *e* per TD feature *f* were summed up for each time step *i*, in order to maintain the influence of each electrode proportionally. This is shown in Equation (1) with $n_{e}$ = 8 for any direction dir. Thus, the feature stream of each direction, was reduced from 32 to 4 features:

(1) $$x_{f,dir}\left[i\right]=\sum_{e=1}^{n_{e}}x_{e,f,dir}\left[i\right].$$

Further on, each feature stream is normalized for itself between 0 and 1. Since each TD feature has a different unit, this step provides equal weight to the features. Equation (2) illustrates this step, where $max(x_{f,dir})$ presents the maximum value of each feature *f* and $x_{min}$ is chosen to be the mean of the recorded rest states of all training sequences:

(2) $$x_{f,dir}^{\prime }\left[i\right]=\left(x_{f,dir}\left[i\right]-x_{min}\right)/\left(max\left(x_{f,dir}\right)-x_{min}\right).$$

Finally, the four features ($n_{f}$ = 4) are summed up at each time step *i* and again normalized to be between 0 and 1, in order to get a maximum label of 1 for each direction (c.f. Equation (3)):

(3) $$label_{dir}\left[i\right]=normalize\left(\sum_{f=1}^{n_{f}}{x^{\prime }}_{f,dir}\left[i\right]\right).$$

Based on these equations, different labeling strategies can be realized. In our previous work [2,42], we used a binary labeling method, which is considered as the baseline for evaluation. In this binary labeling method, samples of the training set in which the activity threshold was exceeded are labeled as ±1 for the respective direction. Accordingly, “inactive” samples as well as samples of a non-active direction were labeled as 0. It has to be noted that even when the labels for the mapping are binary, the output of the decoder still allows for continuous signals, when a regression method is used.

Binary labels ($label_{dir}\left[i\right]\in \left\{-1,0,1\right\}$) are simple to realize but in contrast to continuous labels ($label_{dir}\left[i\right]\in \left\{x\in Q\;|\;-\;1\leq x\leq 1\right\}$), they can not represent the continuous change of the EMG signal.

Additionally, the effect of in- and excluding the transient phase of the EMG signal was investigated. Therefore, data either included the transient phase or relied purely on the steady-state phase of the EMG signal. The first 1000 samples were discarded in case of steady-state labeling. In total, five labeling strategies were evaluated. All labels were derived from the EMG data of the same three training data sets per subject. From the total of 3000 available samples per direction and repetition, each method took 2000 samples into account. The sample selection was dependent on the particular method. The label strategies were realized as followed:

**(A)**  Binary label, including the rising transient phase of the EMG signal in the activ label;
**(B)**  Continuous label, including the rising transient phase of the EMG signal in the active label;
**(C)**  Binary label, using only the steady state of the EMG signal (excluding transient phases);
**(D)**  Continuous label, using only the steady state of the EMG signal (excluding transient phases);
**(E)**  Continuous label, including both transient phases (rising and falling) of the EMG signal.

**Figure 2:**
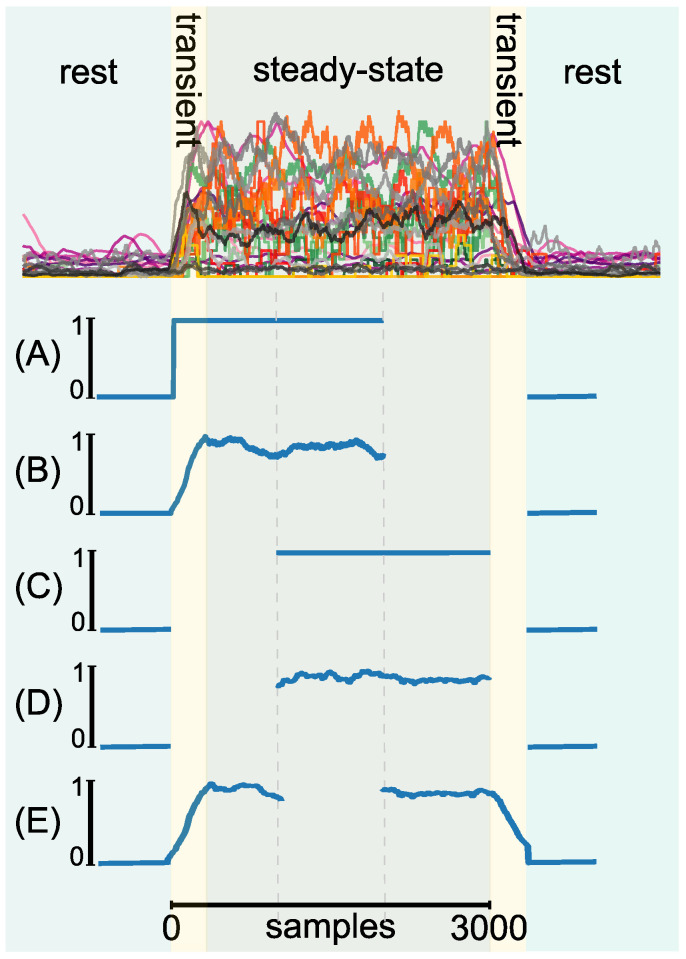
Example for the generated labels. (**A**) Binary label (0 = not active, 1 = active), including the rising transient phase of the EMG signal in the active label. (**B**) Continuous label, including the rising transient phase of the EMG signal in the active label. (**C**) Binary label, using only the steady-state signal (excluding transient phases). (**D**) Continuous label, only using the steady-state part of the signal. (**E**) Continuous label, including the rising and falling transient phases of the EMG signal.

We limited the evaluation to these 5 combinations of labels in order to keep the duration of one experimental session below 90 min. A visualization of the different labeling strategies can be found in Figure 2.

### 2.5. Experimental Procedure

**Figure 3:**
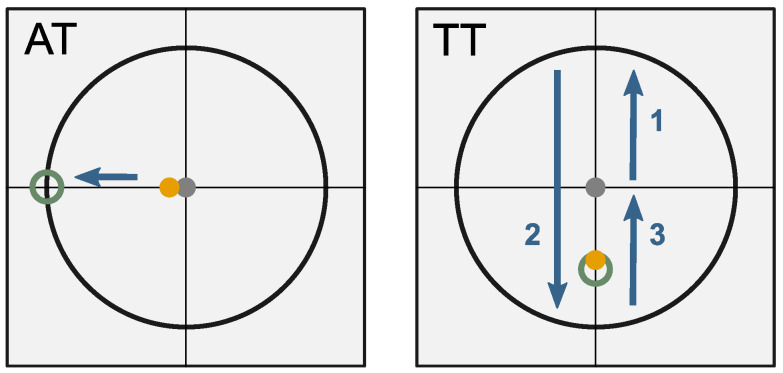
Description of the aiming task (AT) on the left and the tracking task (TT) on the right. The yellow dot displays the cursor controlled by the participants. The gray dot displays the starting point in the middle of the coordinate axes. The green circle is the respective target. During the AT, a target located along the cardinal axis had to be reached as quickly and accurately as possible. During the TT, participants had to track the target which moved with constant velocity along the horizontal or vertical DoF.

An online experiment was conducted to evaluate performance in a 2D task on a computer screen. The decoded EMG signals of the participants were used to control the velocity of a cursor on the screen. Subjects were able to move the cursor in 2 DoF, i.e., up, down and left, right. As one individual predictive model was used for each DoF, simultaneous movements (e.g., diagonal movements) of the cursor were possible. Using this interface, subjects performed two different tasks during the experiments: An aiming task (AT) and a tracking task (TT). The application GUI for both tasks contains crosshairs with an *x*- and *y*-axis. During the AT, a target circle was visible on the screen (c.f. Figure 3, left side). It was placed 400 px from the starting point along one of the main axes. The starting point of each trial was set to the middle of the crosshairs. Subjects were asked to move the cursor as quickly and accurately as possible into the target. The cursor had to stay within the target circle for at least 500 ms to finish the task successfully. After each trial, the cursor was set back to the starting position, where it had to steadily remain for 2 s before the next trial would start. A countdown visualized the 2 s duration before a new trial was initiated. A test sequence involved all four possible directions of a target, presented in random order. One experiment included five test sequences, which leads to a total of 20 trials per AT experiment.

During the TT, the target moved with a constant speed either horizontal or vertical along the axis. The target started in the middle of the coordinate system and traveled in total 1600 px along one DoF (±*x* or ±*y*), including two turns (c.f. Figure 3, right side). Subjects were asked to follow the target and stay within the target diameter as accurately as possible. One test sequence included both DoFs: horizontally and vertically. Five test sequences completed one TT experiment.

Each AT and TT was conducted with all calculated models based on the different labeling strategies. In total, each subject performed five rounds of experiments of AT and TT. The model order was randomized based on a Latin square design over all subjects. The subjects could familiarize to the new control output prior to each experiment round. This included control of the cursor freely on the screen for 30 s, followed by one test sequence, which was not considered in the evaluation.

### 2.6. Subjects

Ten right-handed subjects (1 female, 9 males, age range 21–28 years) took part in this study. None of them reported known neurological diseases or other physical impairments. All of the subjects had prior experience with the EMG-based interface. In particular, they had used the interface in combination with labeling method A as this was used in previous applications. All subjects gave written consent to the procedure, which was explained to them orally and in a written form. The study was conducted according to the guidelines of the Declaration of Helsinki, and approved by the Ethics Committee of the Technical University of Munich, School of Medicine (approval number: 6/14S).

### 2.7. Performance Measurement and Data Analysis

For analyzing the data of the AT following performance measures were used:

- Success rate;
- Completion time, gross, and fine motion time;
- Average and maximum speed during gross motion;
- Path efficiency.

A trial is counted to be successfully finished when the cursor is placed within the target circle for more than 500 ms. If a task could not be finished within 10 s, the trial is considered to be failed. The path efficiency describes the ratio of the shortest distance to the target to the traveled distance of the cursor. The gross motion time is defined as the time from the beginning of the task until the cursor touches the target circle for the first time. The fine motion time starts as soon as the cursor touches the target circle for the first time (end of gross motion) and lasted until the task is finished successfully. Accordingly, the completion time is the sum of gross and fine motion time. The evaluation of the TT was done using the average distance from the cursor to the center of the target. Furthermore, the average travel speed was analyzed.

A statistical analysis was performed on the performance measures gross motion time, fine motion time, completion time, path efficiency, as well as on the performance measures of the TT. Thereby, the following hypothesis should be proofed:

**Hypothesis 1(H_1_).**  *The different labeling methods do not influence task performance when using the EMG-based interface in a 2D task*.

The mean value of each subject per labeling approach and performance measure was used for this analysis to obtain a more expressive result. Since the data was not normally distributed, a Kruskal–Wallis (KW) test was used for statistical analysis. A pairwise Wilcoxon test with Bonferroni correction was used as a post hoc test. The effect size *r* was estimated through the equation given by Rosenthal, 1991 [43]. One sample of the TT in method D showed a value more than twice as the mean, which was not explainable. This value was identified as an outlier and eliminated for further analysis. In addition, a questionnaire was made after each experiment to gather information on the subjects’ confidence in controlling the cursor.

## 3. Results

**Figure 4:**
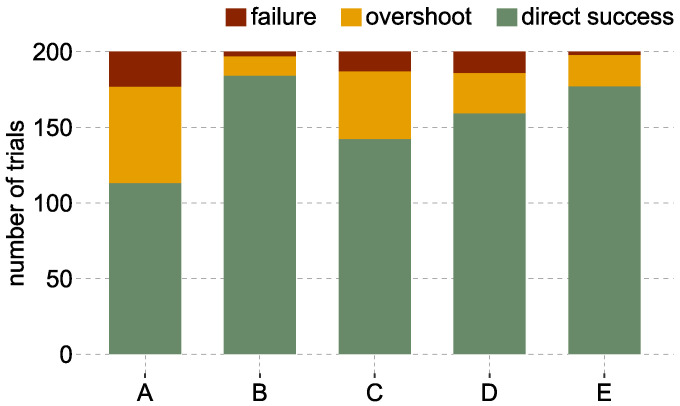
Overshoots over the whole experiment. For each labeling method A–E, the bar plot illustrates how often the trials were finished directly by moving the cursor straight into the target circle (green), a target was overshot before finishing the trial (orange), or the trials failed (red).

In total, 200 trials were performed per labeling method across all subjects during the AT. The highest success rate was reached by the labeling method B with 98.5%, and E with 99% (c.f. Table 1). Both methods are based on the new labeling approach and include a transient phase. Methods C and D show success rates of about 93%, while the baseline method A has a success rate of 88.5%. Most failures occurred during the fine motion time independent of the method (A: 19/23, B: 3/3, C: 12/13, D: 12/14, E: 1/2). Gross motion failures occurred rather rarely. Figure 4 illustrates the failed trials, successful trials with overshoots, and trials which could be finished directly (without overshooting). It can be observed that subjects were able to finish the AT in 92% of the cases without any overshoots using method B. Subjects were able to finish the AT without overshoots in 88.5% of the trials with method E, while method A shows 56.5% of the trials without overshoots.

**Figure 5:**
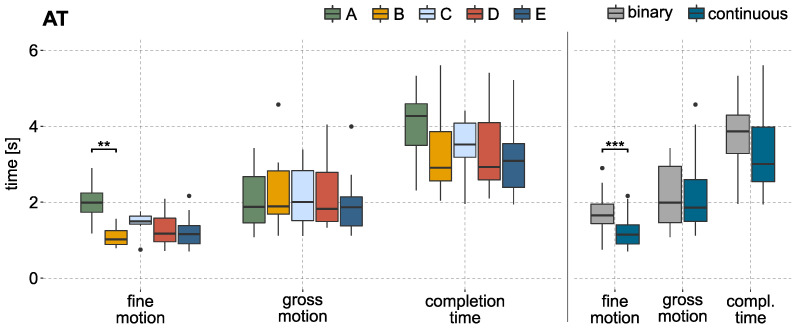
Results of completion time, fine motion, and gross motion time over all subjects during AT, based on the mean values of each subject per labeling method. (**Left**): boxplots of the needed time sorted by labeling method. The statistical analysis identified a significant effect in fine motion time between method A and B. (**Right**): boxplots of needed time sorted by methods based on binary and continuous labels. Binary includes results performed with model A and C; continuous includes results from B, D, and E. The KW test could identify a significant effect for the fine motion time. ‘•’ indicates outliers; ‘*’ indicates statistical significance (‘*’ *p* < 0.05, ‘**’ *p* < 0.01, ‘***’ *p* < 0.001).

Figure 5 illustrates the completion time, gross motion, and fine motion time for successfully finished trials of all subjects. For illustration, the mean values per labeling method and subject are used, analogous to the statistical analysis. The gross motion time shows stable results over the different methods. A significant effect could not be identified. In a direct comparison between all methods using continuous labels (B, D, E) to the methods based on binary labels (A and C), no significant effect of the gross motion time could be found either. The results of this comparison can be seen in Figure 5 on the right side.

In contrast to the gross motion time, the fine motion time shows stronger variation between the methods. A significant effect can be reported by the KW test (*p*-value < 0.01). The post hoc test showed that it took significantly longer to finish the fine motion part when using the binary model A compared to model B (*p*-value < 0.01, r=0.66), which is based on continuous labels. A direct comparison of methods using binary and continuous labels shows a significant effect as well (*p*-value < 0.001, r=0.80).

The analysis could identify a significant effect of the path efficiency during gross motion. The KW test identified methods based on binary labels with an average path efficiency of less than 79% to be less efficient (*p*-value < 0.05, r=0.52) compared to the methods based on continuous labels (path efficiency of B,D,E > 83%). These results can be found in Table 2.

**Figure 6:**
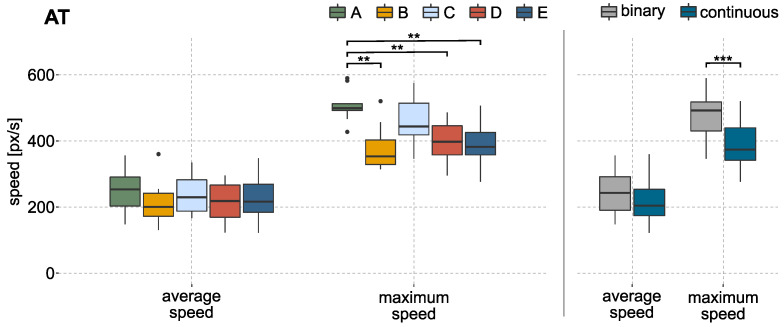
Results of average and maximum speed over all subjects during AT, based on the mean values of each subject per labeling method. (**Left**): boxplot of the average speed and maximum speed in px/s during the gross motion section of the AT. A significant difference was identified for maximum speed (A-(B, D, E) by the KW test. (**Right**): boxplot for the average and maximum speed during the gross motion for methods based on binary and continuously labeled data. A significant difference was identified for maximum speed. ‘•’ indicates outliers; ‘*’ indicates statistical significance (‘*’ *p* < 0.05, ‘**’ *p* < 0.01, ‘***’ *p* < 0.001).

When comparing the cursor speeds during the AT, differences can be observed for the used methods (c.f. Figure 6 on the left side). The maximum cursor speed during gross motion was reached by method A followed by method C, both with an average maximum speed of more than 400 px/s. The cursor would reach a velocity of approximately 600 px/s, if the output of the predictive model is 1. The KW test identified a significant difference in maximum speed. The baseline method A shows an effect when compared to the methods based on continuous labels (B, D, E). A faster maximum cursor speed can also be reported (*p*-value < 0.001, r=0.93) in a direct comparison between methods using binary or continuous labels. The results are visualized in Figure 6 on the right side.

**Figure 7:**
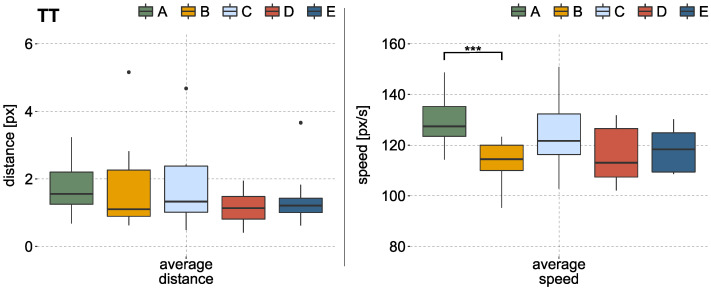
Results of the TT over all subjects, based on the mean values of each subject per labeling method. (**Left**): boxplot of the average distance from cursor to target. The total distance traveled per trial was 1600 px. There was no significant effect between the methods. (**Right**): boxplot of the average travel speed of the cursor. The target moved with a constant velocity of 100 px/s. A significant effect was identified between method A and B by the KW test and the post hoc test. ‘•’ indicates outliers; ‘*’ indicates statistical significance (‘*’ *p* < 0.05, ‘**’ *p* < 0.01, ‘***’ *p* < 0.001).

During the TT, no significant effect on average distance to the target could be identified. The left side of Figure 7 illustrates these results of the TT over all subjects. The statistical analysis obtained a significant difference in average speed during the TT (*p*-value < 0.01, r=0.66). Hence, labeling method A showed a higher average speed compared to method B, which is based on continuous labels. Results can be seen in Figure 7 on the right side. While the average speed of method A lies at about 130 px/s, methods using continuous labels show mainly values lower than 120 px/s. The target moved with a constant speed of 100 px/s.

Subjects had to rate their ability to control the cursor on a scale from 0 (very bad control) to 20 (very good control) after performing each labeling method. Method B reached on average the best rating with 16.3 ± 3 points, while method A was rated worst with 13.6 ± 3.2 points. The results of the questionnaire can be found in Table 2.

## 4. Discussion

In this work, we introduced a new labeling approach to generate continuous labels for proportional electromyographic control. The continuous labels originated from the preprocessed EMG data, recorded during a simple data acquisition procedure. No additional sensors were used to generate the labels. Since the labels were created directly from the EMG feature stream, they reflect the intensity of the muscular activity provided by the participant. By using the EMG data without additional sensors, the challenge of synchronizing external values to the proportional EMG signals as described in Raghu et al. [29] can be avoided. Mismatches that may occur from tracking a visual stimulus during the training procedure e.g., through an incorrect activity level or a delayed muscle contraction can be excluded with this method. The simple training procedure specifies just the category of the label (in this case, the direction) as well as the time to gather enough data. No ramp or MVC levels had to be reached. The predefined activity threshold enables to coincide the onset of the muscular activity with the onset of the labels, including the transient phase. Thus, no additional time delay occurs between the EMG signals and the labels during the training. The time delay given by the system, to transfer and process the data, is the only delay present. However, this delay is inherent to the system and is present during training but also during the usage of the interface.

The realized EMG-based interface achieved success rates between 88.5% and 99% during the aiming task. In the literature it has been shown that aiming tasks are viable to test the performance of EMG-based interfaces [44,45]. In Scheme et al. [44] e.g., two proportional control schemes where compared using a Fitts’ Law test, which presents a special type of an aiming task. Proportional control was added here as a post-processing step to the classification. Since the experimental design differs from our approach (target distances, target and cursor sizes, and simultaneous control), the results are not directly comparable, however a good estimate was given for task performance with EMG-based interfaces during aiming tasks. In Scheme et al. [44], a success rate of up to 96% could be reached. Kamavuako et al. [45] used as well a Fitts’ Law test to measure performance and reached success rates of about 91% with a surface EMG approach and 96% in a combined approach of surface and intramuscular EMG. The success rate of our study (between 88.5% and 99%) lies in a similar range as the results achieved in the mentioned publications. This reveals that our approach provides, generally, a useful control output.

**Figure 8:**
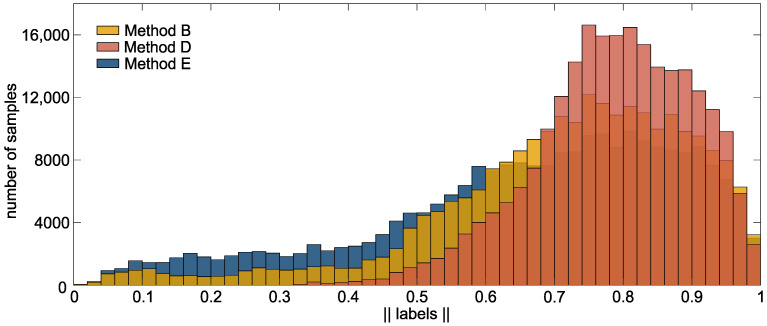
Histogram of the continuous labels. Shown is a histogram of the generated labels of the methods using continuous labels B, D, and E. The data includes the labels between 0 and 1 of all subjects and directions (0 is excluded).

A success rate of up to 99% (B: 98.5%, E: 99%) could be achieved by methods based on the new introduced labeling approach. It is noteworthy that the two continuous labeling methods that include the transient phase (B and E) performed best. Exclusion of transient phases in method D led to a drop in success rate to 93%. Here, the result is comparable to method C (93.5%), a method based on binary labels which also excludes the transient phase. Figure 8 illustrates the histogram of the labels generated by the new labeling approach. The effect of in- or exclusion of the transient phase on the labels can be observed. Methods, including the transient phase, contains more variability in the labels. As an example, in method B and E, 11 % and 21 % of the labels have a value below 0.5. These are considerably more as in method D (1.5 %). We assume that the label-variability, given by the transient phase, improves the control accuracy. This statement is supported by the fact that failures in method D occurred mainly during fine motion (c.f. Table 1), in which a precise commanding is needed to reach the target. Including the transient phase allows for more variability in EMG control while maintaining the simple training procedure. The short transient phases that are required to reach the steady state signal already provide enough variability in the EMG signal to improve the level of proportional control, compared to using steady state data only.

Compared to method B, method E includes two transient phases: the rising as well as the falling flank of EMG data. The success rate of both models shows similar results. Further, a significant effect between these models could not be identified. This indicates that considering the second flank has little effect on task performance. The experience from our prior studies with people suffering severe muscular atrophy showed that it is often a problem to relax the muscles abruptly after voluntary muscle contraction. This might influence the falling EMG signal during training. Therefore, we hypothesize that in our method, no advantage is given by including the falling flank in the training data.

When comparing methods based on continuous labels with methods based on binary labels, differences in success rate, fine motion time, and maximum cursor speed can be observed. Trials performed with a predictive model based on continuous labels show a higher success rate as methods based on binary labels. The baseline method A showed, with 88.5% success rate, the lowest value for all methods. On closer inspection, the effect can be attributed to the fine control of the cursor. While the gross motion time is almost equal over all methods, the outcome of the fine motion time varies. This means that the cursor could be moved correctly in the direction of the target and reach it, however the cursor could not always be moved precisely into the target circle. The statistical analysis confirmed that the fine motion could be controlled more precisely with the methods based on continuous labels. We assume that better fine motion indicates better proportional control as the ability for slow and precise commands is required to place the cursor inside the goal area.

The analysis of the cursor speed showed that a binary labeling strategy leads to faster maximum cursor commands during the gross motion. The active EMG data was labeled with 1 during the data acquisition when a binary label is applied. The generated labels of the newly-introduced approach (c.f. Figure 8) are spread between 0 and 1. This leads to a slower control output, which in turn leads to the ability to control the output more precisely. Although, the maximum cursor velocity during the AT is faster for methods based on binary labels, the average speed did not increase and the gross motion time did not decrease for these models. However, the path efficiency showed that the traveled path was more efficient in methods based on continuous labels (PE for binary labels < 79%; PE for continuous labels > 83%). The average PE of methods based on continuous labels correspond to those found in the literature. A PE of up to 82% was reported in [44] during a Fitts’ Law test. Kamavuako [45] reported a PE of up to 87% in the Fitts’ Law test, which was performed by nine subjects. Both realized the EMG control by the use of a proportional classifier. In Ameri et al. [14], a support vector machine was used to achieve simultaneous and proportional control. Here, a PE of up to 85% could be reported during a 2D aiming task for one subject with transradial limb deficiency and about 76% for 10 healthy subjects during a 3D task.

A detailed inspection shows that especially method A causes differences to the methods with continuous labels. Besides the lower success rate, method A also produces an effect in fine motion time and maximum cursor velocity. Method C, which presents a method that excludes the transient phase, does not cause these effects. This reveals that the inclusion of the transient phase to the binary label further increases the agility in the prediction. Thus, model A appears most agile at the cost of less accuracy.

These findings can also be observed for the tracking task. Higher average speed was achieved when method A was active. A statistical effect was found between A and B. Method B shows the lowest average speed, which is also closest to that of the moving target. Nevertheless, the results of the average distance to the target indicates that an equivalent performance could be reached by all methods. Apparently the selected speed of the target of 100 px/s can be tracked equally well with all five labeling methods. We suggest further tests with varying velocities of the target to investigate more detailed effects regarding task performance during tracking.

With the results shown above, the Hypothesis 1 (H_1_) can be partially rejected, since the task performance of fine motion and cursor speed are influenced by the different labeling methods, especially when distinguishing between continuous and binary labeling methods.

The evaluation of the different labeling strategies indicates that a continuous label leads to more precision during fine motion control. The control of prostheses or assistive robots often requires precise proportional control in order to master tasks of daily living. Therefore, our method can potentially help to improve the control of assistive devices. The results of the questionnaire indicate that the participants preferred a precise control as given by method B over a reactive control as given by method A for the given task. This is expected, as the performance is worse using method A, with participants rating this method lower. The assessment of the participants supports the results of the quantitative measures, such as the success rate and fine motion.

A high frustration level due to a lack of control is one of the reasons to reject an assistive device [26]. An increased confidence about the control may therefore increase the acceptance of the assistive device. However, the findings about the sensitive control given through a binary label and the inclusion of the transient phase, as given in method A, are of interest to systems using shared control. Assistive devices with an integrated shared control algorithm support the user during complex tasks of daily living. Operators command the device roughly along a task. Precise movements and fine motions are then supported by the system [46], which makes the task easier for the user. Here, the advantage of more reactive control may outweigh that of precise control because deficits in precision can be compensated by shared control. A detailed analysis of the labels during tasks of daily living and with and without shared control would be necessary to confirm that assumption.

Prior research shows that the presented EMG-based interface can be used in 3D applications with a robotic system [38]. Baseline method A was used as labeling method to perform tasks of daily living with an assistive robot. The interface allows a proportional and simultaneous control of the robotic device in 3D. Although, in this investigation the labeling methods were evaluated in a 2D task on a screen, we assume that the results may be valid for proportional and simultaneous control in 3D.

As sensor shift and drift are still problems in EMG-based control, techniques of online adaptations are useful to update the predictive model during the application if control commands are not fitting anymore [47]. The introduced approach provides continuous labels that can be created during the usage of the interface. It is neither dependent on a defined training procedure nor on additional sensors to generate the continuous labels. Thus, the training data can be gathered during the application and interrupting the application is avoidable. This makes the introduced approach ideal to update the predictive model online with continuous labels.

## 5. Conclusions

This work presents a new labeling approach for continuous labels used in proportional EMG control. Labels are directly extracted from EMG features calculated during data acquisition. The method allows for a simple training procedure and still covers the variability of the EMG signal by including the transient phase of muscle contraction during training. No additional sensors were needed and the method ensures a minimum time delay between the EMG training data and the generated labels. Furthermore, mismatches due to wrong contraction levels are avoided. Thus, this labeling method presents an ideal method for people with physical disabilities, especially if additional sensors are not an option. However, this work does not provide a comparison between the introduced approach and other labeling strategies, such as tracking a visual stimulus or additional sensors. At this point we can also not make any statement on the control with prosthesis or other EMG applications. Further studies are mandatory to verify if the introduced method can be used or even improve e.g., prosthesis control.

We investigated the effect of the introduced labeling approach on task performance when using an EMG-based interface. To quantify the effect, participants performed tasks with five different mappings based on the same training data, however with different labeling methods. The five different methods included continuous labels, a baseline method with binary labels, as well as variations thereof with in- and excluding the transient phases of the EMG signal. In total, three variations of the new labeling approach (called: B, D, and E) were compared with two variations of a binary labeling strategy (called: A and C). The evaluation showed that the methods based on continuous labels and including transient and steady-state phases led to high success rates of 98.5% with B and 99% with method E, compared to methods based on binary labels, which showed success rates of 88.5% with A and 93.5% with method C. Differences between the methods were particularly evident during fine motion. A significant difference could be identified between methods based on binary and continuous labels, as well as between method A and B. This indicates improved fine motion capabilities with methods using continuous labels and a higher command accuracy in comparison with methods using binary labels.

To conclude, the introduced continuous labels in combination with the transient phase (corresponding to labeling method B) presents an efficient way to map muscular activity to a proportional control input with good command accuracy. The baseline method, which uses binary labels, proved to be more reactive and less accurate. In applications where no precise control is required, as e.g., in systems providing shared control, these strategies may be advantageous. In future, the introduced approach must be deployed with a robotic system in 3D to confirm the achieved results with the intended system of use. Furthermore, the approach must be evaluated with actual users suffering from severe muscular atrophy, where this labeling method is of particular interest, as additional sensors are not an option for generating appropriate labels for this group of users.

## Figures and Tables

**Table 1:** Success rates and failures over all subjects during the AT.

		A	B	C	D	E
	success rate	88.5%	98.5%	93.5%	93%	99%
Complete trial	failure	23	3	13	14	2
Fine motion	failure	19	3	12	12	1
Gross motion	failure	4	0	1	2	1

**Table 2:** Path efficiency and results of the questionnaire. The table show the path efficiency during the gross motion section of the AT over all subjects. The questionnaire asked the subjects how well they could control the cursor during each experiment. They could rate from 0 to 20, where 0 represented bad control of the cursor and 20 represented a perfect control.

			A	B	C	D	E
path efficiency (PE)		mean in %	76.7	83.1	78.9	83.5	84.0
		±sd	17.7	15.0	17.9	14.2	14.4
questionnaire: How good was the control of the cursor?		mean (0–20)	13.6	16.3	15.3	15.4	15.9
		±sd	3.2	3.0	1.9	2.3	2.2

## Abbreviations

The following abbreviations are used in this manuscript:

EMG	Electromyography
DoF	Degree of Freedom
GP	Gaussian Process
AT	Aiming Task
TT	Tracking Task
TD	Time Domain
PE	Path Efficiency
KW	Kruskal Wallis
GUI	Graphical User Interface
